# Effects of landscape characteristics on genetic structure and gene flow of *Camellia chekiangoleosa* at Laohunao Nature Reserve

**DOI:** 10.3389/fpls.2025.1713503

**Published:** 2026-04-13

**Authors:** Zheng He, Kaiming Zeng, Yue Deng, Bicai Guan

**Affiliations:** 1 Technology Innovation Center for Land Spatial Ecological Protection and Restoration in Great Lakes Basin, MNR, Jiangxi Institute of Land Space Survey and Planning, Nanchang, China; 2 Key Laboratory of Poyang Lake Environment and Resource Utilization Ministry of Education, Nanchang University, Nanchang, China

**Keywords:** *Camellia chekiangoleosa*, genetic diversity, landscape genetics, isolation-by-environment, landscape pattern

## Abstract

Understanding how landscape features influence gene flow in natural populations is a central goal of landscape genetics. In this study, we evaluated the status of multiple populations of *Camellia chekiangoleosa*—a provincially protected plant in Jiangxi Province with significant ecological and economic value—in the Laohunao Nature Reserve. We then performed an analysis of their genetic diversity and spatial genetic structure to inform the development of science-based conservation strategies. The findings indicated that the genetic diversity of *C. chekiangoleosa* was on par with that of the majority of other species within the genus. Sub-populations exhibit a certain degree of genetic differentiation (*F*_ST_=0.065). The application of STRUCTURE and principal component analysis (PCA) unveiled a distinct pattern of geographical clustering across the 9 sub-populations. Multiple matrix regression with randomization (MMRR) revealed that both environmental isolation and geographical isolation have wielded a pronounced impact on genetic distance. The MEMGENE analysis further discerned spatial genetic structure, which was influenced by the ridges and valleys that demarcate Mount Ruozhushan (RZS), Mount Zhushan (ZS), and Mount Shitoushan (STS). Mountain ridges exerted a more pronounced influence on genetic differentiation compared to valleys. In summary, this study underscores that topographic features play a pivotal role in shaping the spatial genetic structure of *C. chekiangoleosa*.

## 1 Introduction

Landscape genetics offers a robust framework for forecasting how particular landscape impediments impact gene flow and diminish genetic diversity (Chau et al., 2019). Both the topographical features of the landscape and the life history characteristics of species engage in an interaction-frequently in unforeseeable manners—to either expedite or impede genetic exchange among populations (Slade et al., 2013; Decocq et al., 2021). The extent and pattern of population divergence are predominantly dictated by the actual gene flow (Slarkin, 1985). Geographical isolation assumes a pivotal role in fostering divergence and giving rise to distinct phylogeographic discontinuities (Zhang et al., 2020). Physical barriers, such as mountain ranges and glaciers, can impede gene flow, thereby resulting in genetic differentiation among isolated populations (Qiong et al., 2017; Chi et al., 2019). An expanding corpus of research has substantiated that factors encompassing geographical separation, forests, urban agglomerations, rivers, and mountain chains can mold gene flow in a variety of ways (Wu et al., 2019; Nilsson et al., 2010; Curto et al., 2015). At a fine-grained spatial scale, restricted gene flow is regarded as a fundamental catalyst for the spatial genetic architecture within populations (Hardy et al., 2006). Via landscape genetics, we can evaluate the relative contributions of geographic distance (isolation-by-distance, IBD) and environmental variability (isolation-by-environment, IBE) to genetic connectivity and differentiation.

*Camellia chekiangoleosa*, an indigenous woody oil-bearing plant in China, is highly esteemed for its resplendent, oversized blossoms, superior-quality seed oil (Zhou et al., 2012), and its invaluable role as a genetic reservoir for the enhancement of other camellia species (Min and Bartholomew, 2007; Xie et al., 2011; Guo et al., 2010; Huang et al., 2013; He et al., 2020; Chen et al., 2021). During the meticulous field surveys, a comprehensively spread wild population of *C. chekiangoleosa* was unearthed within the Laohunao Nature Reserve of Jiangxi Province. This population has been fragmented into nine diminutive sub-populations by the ridges, along which wind power facilities have been erected. It thrives in broad-leaved forests situated at an altitude of roughly 1000 meters above sea level, in the vicinity of the mountain peaks. Landscape heterogeneity, encompassing topographical disparities and habitat gradients, frequently exerts a profound influence on gene flow within natural ecosystems (Tsuda et al., 2010). Moreover, the substantial fruits of *C. chekiangoleosa* are consumed and disseminated by animals, a phenomenon that may expedite gene flow among populations. Landscape genetics typically delves into how terrain characteristics mold genetic connectivity. For example, the landscape structure impacts plant dispersal and propagation, consequently modifying gene flow and the spatial distribution of genetic variation (Kamm et al., 2009; Kramer et al., 2011). In *Salix humboldtiana*, the genetic structure emerges from intricate interactions among topography, climate, and river salinity (Hernández-Leal et al., 2022). While rivers and valleys have been shown to foster gene flow in certain species (Nilsson et al., 2010; Murray et al., 2019; Liang et al., 2019), studies on *Passiflora* have indicated that rivers can also function as barriers to genetic exchange (Caze et al., 2016).

Building upon a preliminary assessment of genetic diversity and population structure in *C. chekiangoleosa*, this study utilizes landscape genetic methodologies to explore the degree to which topographic elements, especially valleys and ridges, influence gene flow and fine - scale genetic structure. By quantifying the impacts of geographical distance and environmental configuration on genetic differentiation, we aspire to offer a solid scientific foundation for the conservation of *C. chekiangoleosa* populations.

## 2 Materials and methods

### 2.1 Collection and preservation of plant materials

**Figure 1:**
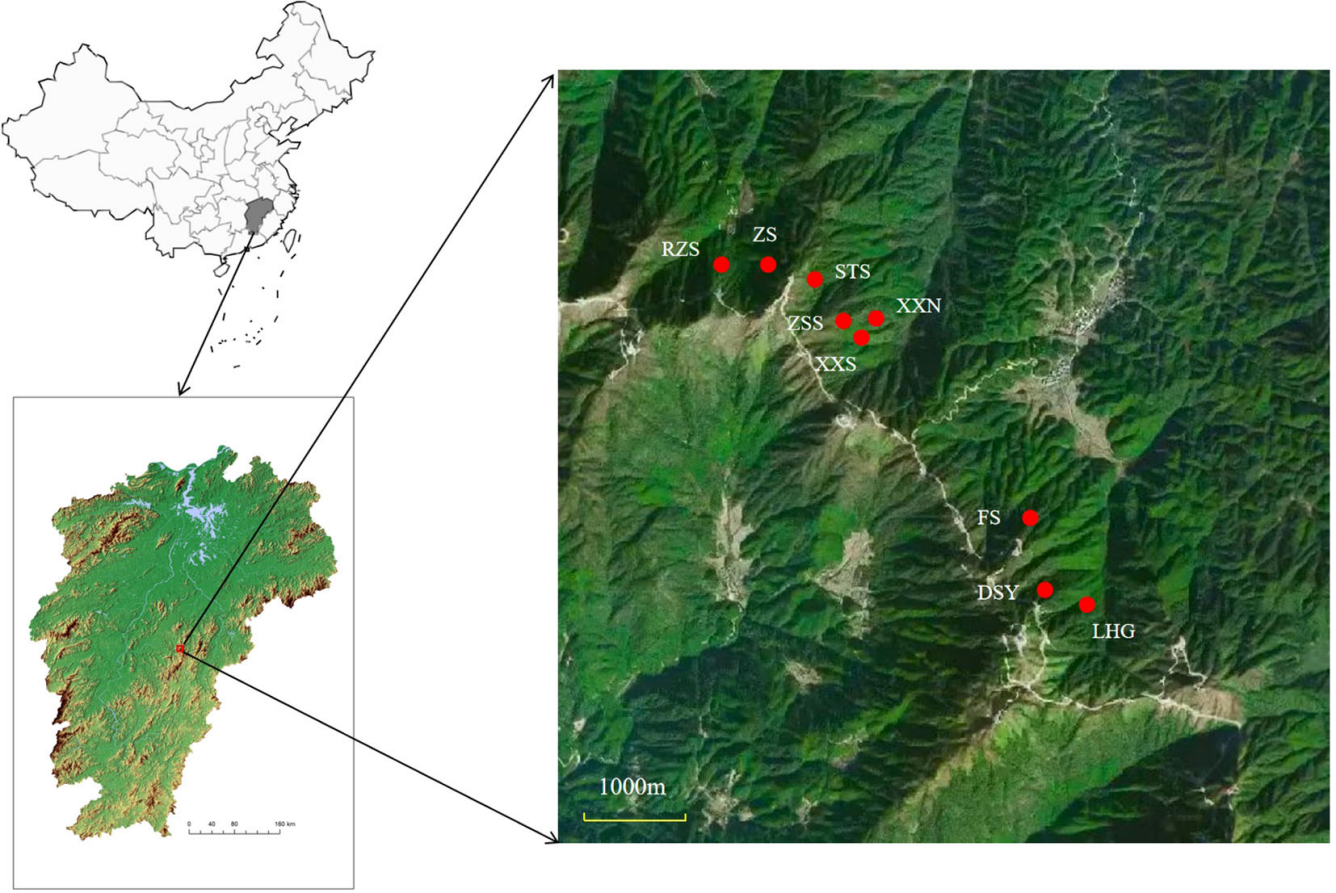
The distribution map of 9 *Camellia chekiangoleosa* sub-populations at Laohunao Nature Reserve, Jiangxi Province, China.

In accordance with the relevant regulations regarding wild plant sampling in China, during the summer of 2022, samples of *C. chekiangoleosa* were procured from 9 sub-populations (abbreviated as XXN, XXS, ZS, ZSS, RZS, STS, LHG, DSY, FS) within the Laohunao Nature Reserve (as presented in **Table 1** and **Figure 1**). These 9 sub-populations, geographically segregated by valleys or ridges, are all situated within the same mountain range, specifically in evergreen broad-leaved forests at similar elevations. The soil across these areas is uniformly classified as yellow-brown earth. From healthy plants, clean and disease-free leaves were carefully collected and placed into tea bags, followed by desiccation using silica gel. Subsequently, the samples were promptly transported back to the laboratory and stored in an ultra-low temperature refrigerator at - 80°C for subsequent DNA extraction.

**Table 1:** Geographical information of 9 sub-populations *Camellia chekiangoleosa*.

Site abbreviations	Sub-populations	Longitude	Latitude	Altitude(m)
XXN	Mount Xiaoxi north	115°59′35.05″	27°13′13.96″	1130
XXS	Mount Xiaoxi south	115°59′31.32″	27°13′11.58″	1082
ZS	Mount Zhushan	115°59′6.78″	27°13′21.92″	1242
ZSS	Mount Zhangshushan	115°59′36.06″	27°13′9.44″	1094
RZS	Mount Ruozhushan	115°58′58.21″	27°13′21.27″	1255
STS	Mount Shitoushan	115°59′16.84″	27°13′21.52″	1156
LHG	Mount Laohugou	116°0′33.89″	27°11′52.41″	1163
DSY	Mount Dishuiya	116°0′26.24″	27°11′55.68″	1207
FS	Mount Fengshan	116°0′19.77″	27°12′20.41″	1142

### 2.2 DNA extraction, library preparation, and sequencing

The Ezup column plant genome DNA extraction kit produced by Shanghai Sangon Biotech Co., Ltd. was used to extract DNA according to the kit instructions. After DNA extraction, used agarose gel electrophoresis to detect DNA quality, and used gel imager to check the electrophoresis results, and stored the extracted DNA in an ultra-low temperature refrigerator at -80 °C. Sent to Beijing Novogene for Genotyping-by-sequence (GBS), and obtained SNPs for further analysis through library construction and data quality control.

In this study, the GBS technology was used to construct a sequencing library of *C. chekiangoleosa*. The library construction process included the following steps: (1) detection of reference genome sequence using silico enzyme digestion and enzyme digestion effect testing; (2) first enzyme digestion using *Mse* I, and second enzyme digestion using a combination of enzymes; (3) detection of DNA fragments by agarose gel electrophoresis, with a recovered fragment length of 300–500 bp; (4) purification of the DNA fragments using AMPure XP beads for qualitative detection, and completion of GBS library construction; (5) after successful library construction, quantification and insert size detection were performed using a Qubit2.0 Fluorometer and Agilent 2100 bioanalyzer; (6) sequencing was performed on the IlluminaHiSeq PE150 sequencing platform provided by Novogene Corporation.

### 2.3 Single nucleotide polymorphisms calling

The raw reads were processed using the fastp software (Chen et al., 2018) to remove adapter information and remove low-quality and unmeasured bases (N), resulting in effective data (clean reads). The clean reads were aligned to the reference genome using the BWA software (Li and Cd-hit, 2006), with parameters set as mem -t 4 -k 32 -M. Subsequently, duplicate reads were removed from the alignment results using the SAMTOOLS software (Li et al., 2009), with the parameter set as sort. The *C. chekiangoleosa* genome sequence was used as the reference genome sequence. The clustering and genotyping were performed using the ustacks program in the Stacks software package (Catchen et al., 2011), with parameters set as -m 3 –M 2 –N 4. Finally, the genotyping results were filtered using vcftools 0.1.13 (Danecek et al., 2011), with the following filtering criteria: elimination of loci with MAF (Minor Allele Frequency) less than 0.05 and loci with a SNP genotyping missing rate greater than 10%, ultimately selecting high-quality SNPs for further analysis.

### 2.4 Analysis of the genetic data

The genetic diversity indices, including the effective number of alleles (*N*_e_), Shannon index (*I*), observed heterozygosity (*H*_o_), and percentage of polymorphic loci (*%P*) were calculated utilizing the GenALEx 6.51 package (Peakall and Smouse, 2012). Additionally, principal component analysis (PCoA) and molecular variance analysis (AMOVA) were performed to elucidate the genetic structure and variation within the dataset. Gene flow (*N*_m_) was calculated based on the genetic differentiation coefficient (*F*_ST_), with the formula *N*_m_=1-*F*_ST_/4*F*_ST_. Cluster analysis was performed using the mixing model of STRUCTURE 2.3.4 (Evanno et al., 2005), and the results were visualized using CLUMPP sampling detection and distruct 1.1. The influence of geographical distance and altitude distance on genetic distance was analyzed using the MMRR package in R (Wang, 2013). We used the ArcGIS 10.6 clipping tool to obtain the raster data of the sampling point rectangular buffer, then we used the MEMGENE package (Galpern et al., 2014) to extract the MEMGENE variable. Additionally, we overlayed the first MEMGENE variable (MEMGENE1) on the resistance surface used to create spatial genetic data by using the add.plot=TRUE parameter. We visualized the level of genetic differentiation across the landscape through the feature vector score of the sample.

## 3 Results

### 3.1 Genotyping-by-sequence data analysis

The original sequencing data of *C. chekiangoleosa* was filtered under strict conditions to obtain high-quality clean data. The sequencing quality was high, the proportion of effective sequences in the original sequences was over 96%, Q20 ≥ 91.32%, Q30 ≥ 80.64%, GC distribution was normal, and the results of library construction and sequencing were qualified. The alignment rate of 119 samples ranged from 95.41% to 98.51% when high-quality sequencing data was aligned with reference genomes. The average coverage depth of assembled genomes ranged from 6.0 X to 16.41 X. The proportion of sites with at least one base coverage was above 4.35%. The alignment results were normal and could be used for subsequent SNP testing. A total of 2,497,326 SNPs were detected in 119 individuals, and 13,463 SNP loci were obtained through data quality control.

### 3.2 Genetic diversity and genetic differentiation

The sub-populations exhibited a number range of 1.853 (LHG) to 1.912 (XXS) alleles (*N*_A_), with an average of 1.891. The effective number of alleles (*N*_E_) ranged from 1.477 (FS, DSY, STS) to 1.493 (XXS), with an average of 1.483. The Shannon’s information index (*I*) varied from 0.425 (LHG) to 0.439 (XXN), with a mean of 0.433. Expected heterozygosity (*H*_E_) ranged from 0.281 (FS) to 0.289 (XXN), while observed heterozygosity (*H*_O_) ranged from 0.402 (STS) to 0.431 (XXS). The average values of *H*_E_ and *H*_O_ for *C. chekiangoleosa* were 0.285 and 0.412, respectively. The percentage of polymorphic loci (%*P*) ranged from 85.33% (LHG) to 91.15% (XXS), with a mean of 89.08% (**Table 2**). The results of AMOVA revealed that the genetic variation of different sub-populations of *C. chekiangoleosa* was 7% (*p* < 0.01) among the population, and the primary variation in *C. chekiangoleosa* was attributed to differences within sub-populations (93%, *p* < 0.01). Therefore, there was greater genetic differentiation within sub-populations (**Table 3**). The number of migrants (*N*_m_) among the 9 sub-populations ranged from 5.803 to 17.780, and the *N*_m_ exceeded 1 (*N*_m_=3.596), indicating that there are varying degrees of gene exchange among the 9 sub-populations (**Table 4**).

**Table 2:** Genetic diversity index of 9 sub-populations.

Sub-populations	N	*Na*	*Ne*	*I*	*Ho*	*He*	*%P*
DSY	13	1.892	1.477	0.430	0.408	0.283	89.19%
FS	13	1.878	1.477	0.427	0.409	0.281	87.75%
LHG	13	1.853	1.483	0.425	0.416	0.282	85.33%
XXN	14	1.901	1.492	0.439	0.422	0.289	90.14%
RZS	13	1.882	1.480	0.430	0.407	0.283	88.16%
XXS	15	1.912	1.493	0.437	0.431	0.288	91.15%
STS	13	1.901	1.477	0.433	0.402	0.284	90.08%
ZS	13	1.906	1.481	0.437	0.405	0.287	90.56%
ZSS	12	1.893	1.483	0.435	0.410	0.286	89.34%
Mean	13.2	1.891	1.483	0.433	0.412	0.285	89.08%

N, Number of samples; *Na*, Number of alleles; *Ne*, Effective number of alleles; *I*, Shannon’s information index; *H_O_*, Observed heterozygosity; *He*, Expected heterozygosity; *%P*, Polymorphism information content.)

**Table 3:** Molecular variance (AMOVA) for *Camellia chekiangoleosa*.

Source	df	SS	MS	Est.Var.	Percentage of Variation(%)	*F*_ST_	*N*_m_	P
Among Pops	8	15200.276	1900.035	68.860	7%	0.065	3.596	<0.001
Within Pops	110	108894.774	989.952	989.952	93%			<0.001
Total	118	124095.050		1058.813	100%			<0.001

df, degrees of freedom; SS, sums of squares; MS, mean squares; Est. Var., estimated variance.

**Table 4:** The number of migrants (*N*_m_) among 9 sub-populations of *Camellia chekiangoleosa*.

	DSY	FS	LHG	XXN	RZS	XXS	STS	ZS	ZSS
DSY	***								
FS	10.105	***							
LHG	8.880	7.688	***						
XXN	6.995	6.407	5.865	***					
RZS	7.523	6.662	6.439	6.640	***				
XXS	6.886	6.244	**5.803**	**17.780**	6.485	***			
STS	8.386	7.505	6.922	7.231	10.331	7.088	***		
ZS	8.090	7.546	6.965	7.365	11.524	7.156	14.000	***	
ZSS	7.359	6.628	6.144	11.978	7.101	11.306	7.872	7.945	***

Bold values represent the maximum and minimum values of gene flow (*N*_m_).

**Figure 2:**
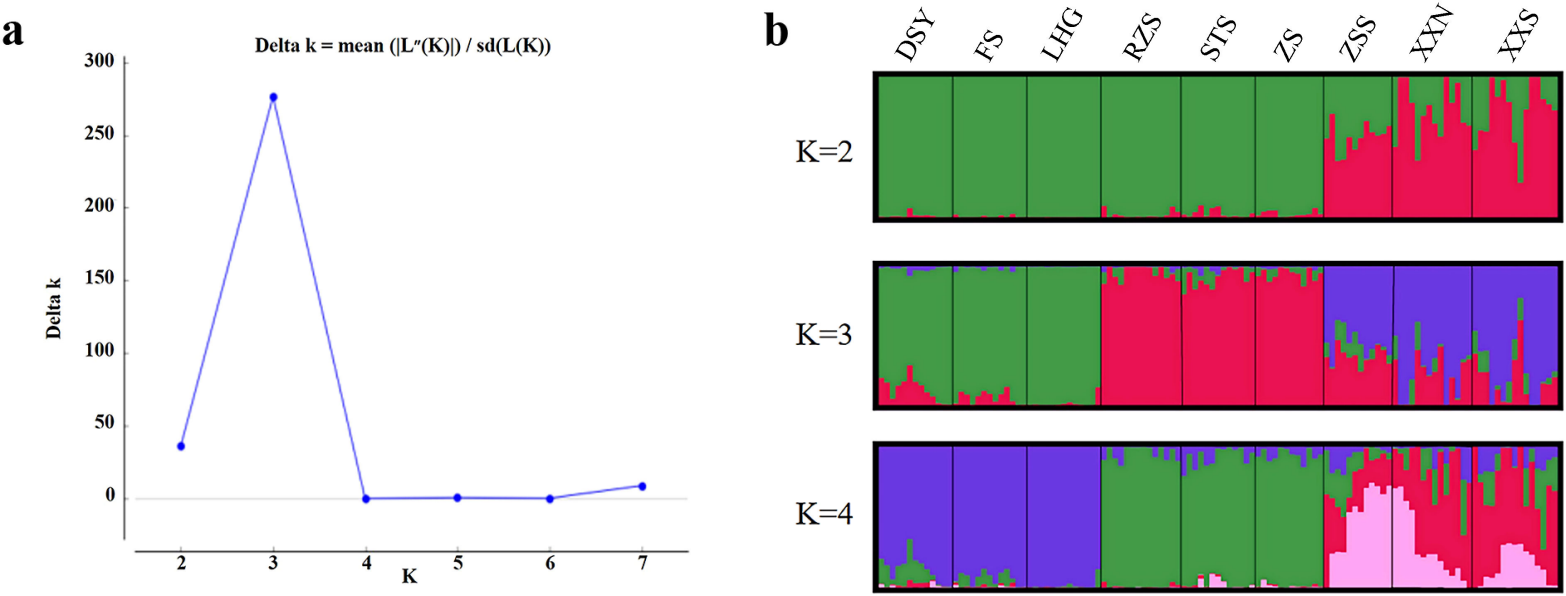
Results of genetic structure analyses in *Camellia chekiangoleosa* sub-populations. **(a)** Relationship between Delta K and K value. **(b)** Summaries of genetic structure analyses when K = 2, K = 3, and K = 4.

**Figure 3:**
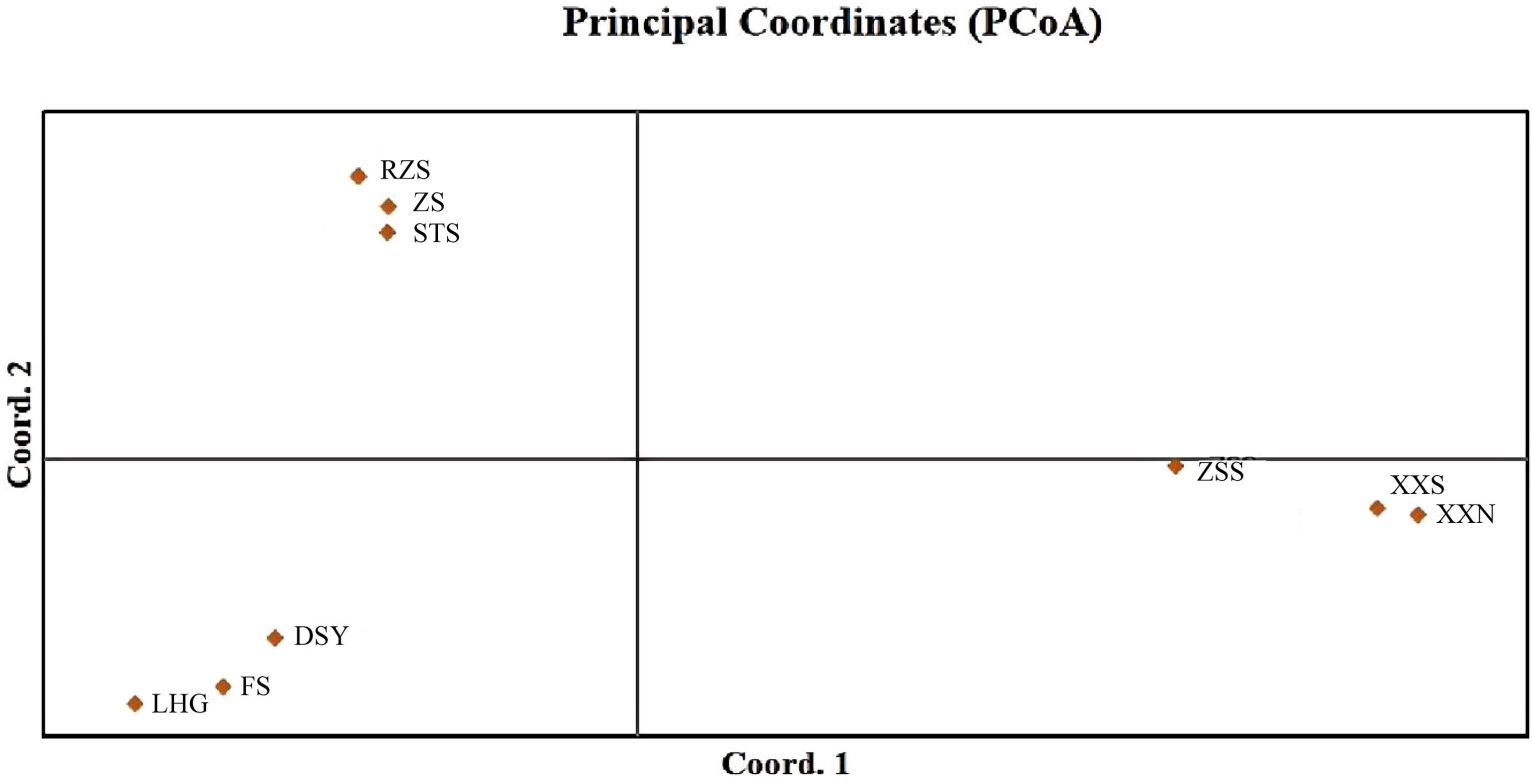
Principal component analyses (PCA) among *Camellia chekiangoleosa* sub-populations. PCA axis 1 explained 29.38% of the variance, whereas PCA axis 2 explained 20.96%. Different colors represented 9 sub-populations.

Structure analyses was conducted on the population data of 13,463 SNPs from the 9 sub-populations. The results obtained from structure harvester indicated that the most suitable clustering was achieved when K = 3 (**Figure 2A**). A considerable number of samples from Mount Zhushan (ZS), Mount Ruozhushan (RZS), and Mount Shitoushan (STS) clustered together. The results indicated that Mount Laohugou (LHG), Mount Fengshan (FS), Mount Dishuiya (DSY) formed a cluster. Structure analyses suggested distinct clusters separating Mount Zhangshushan(ZSS) from South Xiaoxi (XXS) and North Xiaoxi (XXN)(**Figure 2B**). The first principal component (PC_1_), the second principal component (PC_2_), and the third principal component (PC_3_) of the principal component analysis (PCA) respectively captured 29.38%, 20.96%, and 12.47% of the total variance. The PCA divided the 9 sub-populations into 3 groups (**Figure 3**), consistent with the grouping results of Structure analyses when K = 3. Both clustering analyses indicated that there is a geographical clustering phenomenon in the sub-populations of *C. chekiangoleosa* in a small scale.

### 3.3 Spatial genetic structure of 9 sup-populations

**Figure 4:**
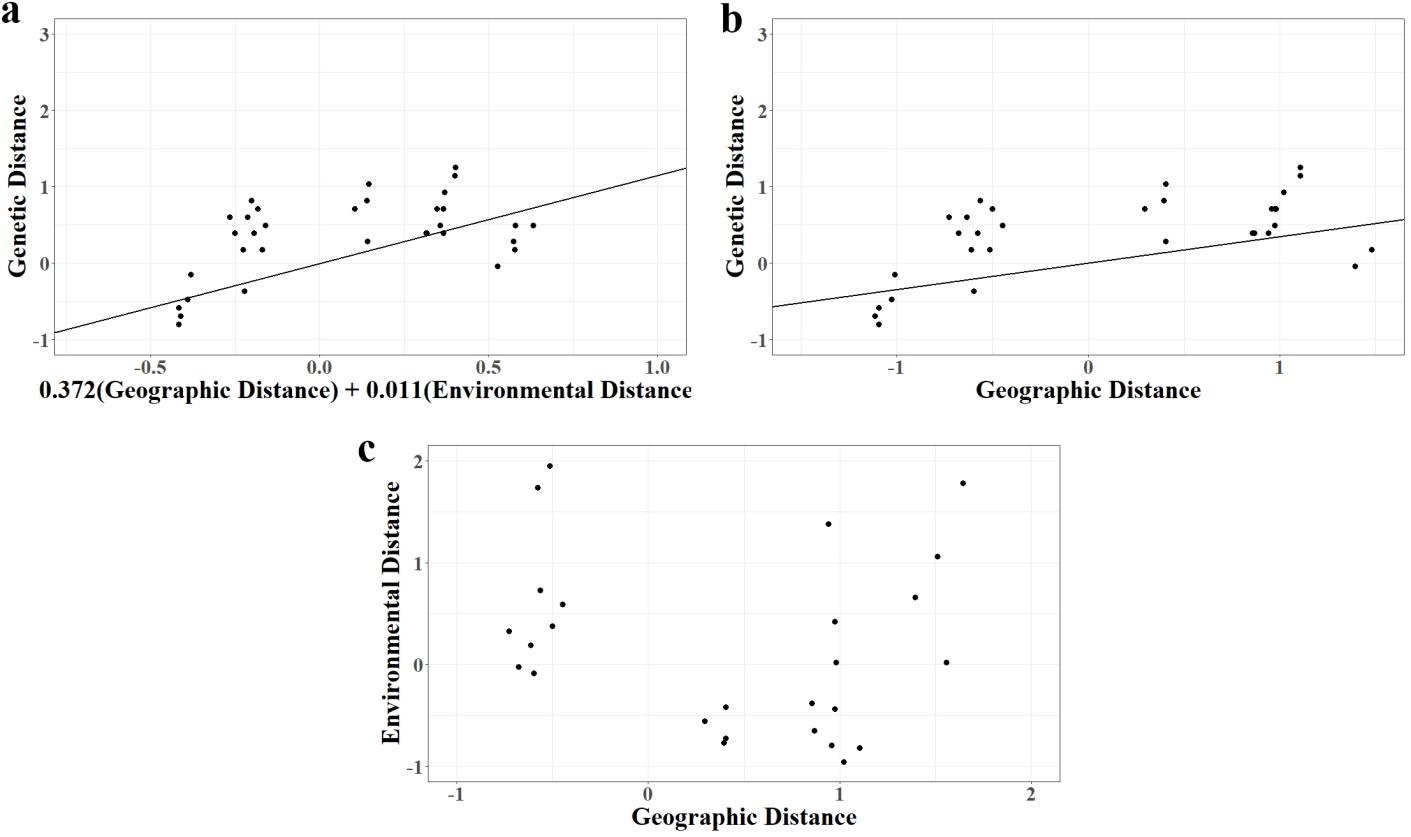
**(a)** Geographical distance and environmental distance effects on genetic distance (R^2^=0.343, *p*=0.008). **(b)** Correlations between geographic distance and genetic distance (R^2^=0.423, *p*=0.000). **(c)** Correlations between geographic distance and environmental distance.

**Figure 5:**
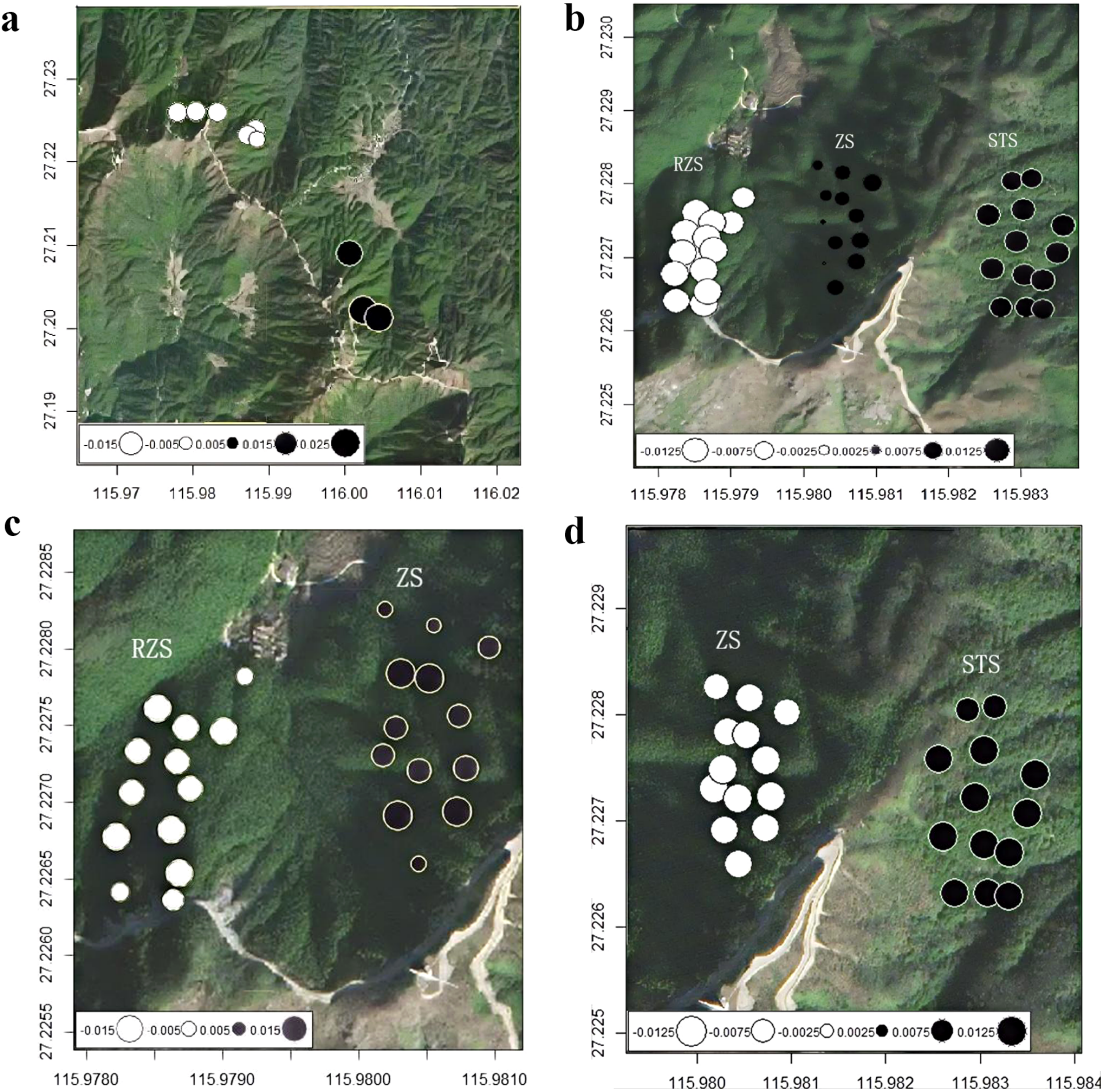
Results of MEMGENE analysis of *Camellia chekiangoleosa*. Size and color of the circles depicted genetic similarity, with large black and large white circles at opposite extremes of the MEMGENE axis. **(a)** 9 sub-populations. **(b)** sub-populations of RZS, ZS, STS. **(c)** sub-populations of RZS and ZS. **(d)** sub-populations of ZS and STS.

Drawing upon the population genetic distance and geographical distance of *C. chekiangoleosa*, a Multiple Matrix Regression with Randomization (MMRR) was carried out. The outcomes of the MMRR analysis indicated that both environmental isolation and geographical isolation have exerted a marked influence on genetic distance(R^2^ = 0.343, *p* < 0.01). The regression coefficient of geographical distance (*β*_D_ = 0.37, *p* < 0.01) outstripped that of environmental distance (*β*_E_ = 0.01, *p* < 0.01) (**Figure 4A**). Additionally, a significant association between genetic and geographical distances was observed (R^2^ = 0.423, *p* < 0.01) (**Figure 4B**), environmental isolation has also had a notable impact on genetic distance. There was no discernible correlation between geographical distance and environmental distance (**Figure 4C**). Circle color and size representing genetic similarity along the first MEM variable axis (explaining 70.7% of overall genomic variation in SNP data), and the second MEMGENE axis explained 29.3% of the genetic variation in SNP data. These two MEMGENE axes explained all of the variability. The proportion of genetic variation explained by spatial structure (R^2adj^=0.032) indicated a relatively weak overall genetic structure. The MEMGENE results of *C. chekiangoleosa* indicated genetic differentiation among 9 sub-populations (**Figure 5A**), and the genetic discontinuity of the 5 representative sub-populations was consistent with the landscape pattern of valleys and ridges. Firstly, RZS, ZS, and STS were separated by ridge and valley (**Figure 5B**). RZS on the left side of the ridge had a different spatial genetic domain from ZS and STS on the right side of the ridge. The first MEMGENE axis and the second MEMGENE axis could respectively explain 59.6% and 40.4% of the genetic variation, with a small proportion of genetic variation (R^2adj^=0.032), but sufficient to identify genetic variation corresponding to landscape features. Secondly, RZS and ZS were separated by a ridge (**Figure 5C**). MEMGENE axis 1 and MEMGENE axis 2 explained 57.1% and 42.9% of genetic variation, respectively, with an explained genetic variation ratio of R^2adj^=0.068. The genetic disruption of RZS and ZS was consistent with the spatial structure of the ridge. Thirdly, ZS and STS were separated by a valley (**Figure 5D**). The first MEMGENE axis score was 100%, indicating that it could explain the maximum difference between the SNP loci of ZS and STS. The interpretation proportion of spatial genetic patterns was R^2adj^=0.012, and the analysis results identified the genetic disruption of ZS and STS on both sides of the valley. MEMGENE axis 1 and MEMGENE axis 2 could explain 59.9% and 40.1% of genetic variation, respectively, with a proportion of genetic variation (R^2adj^=0.016). The analysis results indicated that there was a mixed genetic similarity between the 2 sub-populations on both sides of the valley, without complete genetic discontinuity caused by the valley. In conclusion, the genetic discontinuity among the 9 C*. chekiangoleosa* sub-populations coincided with the landscape distribution pattern, and the influence of ridges on genetic differentiation is greater than that of valleys.

## 4 Discussion

This study delved into the genetic architecture of the *C. chekiangoleosa* population in the Laohunao Nature Reserve. It was found that, when compared with most of its congeneric species, this specific population exhibits a relatively high degree of genetic diversity (Huang, 2013; Peng, 2016; Lai, 2021; Shi, 2022), along with a level of genetic divergence. The utilization of STRUCTURE and principal component analysis (PCA) revealed a discernible pattern of geographical clustering among the 9 sub-populations. The genetic diversity of *C. chekiangoleosa* is in stark contrast to that of its more endangered congeneric counterparts, such as *C. mingii* and *C. debaoensis* (Shi, 2022). Presumably, this diversity is sustained by its reproductive biology, which includes dual pollination mechanisms (wind-and insect-mediated) and efficient seed dispersal by animals, thus promoting a vigorous gene flow (Kamm et al., 2009; Qi et al., 2022; Yan et al., 2022). Such gene flow can alleviate the detrimental effects of genetic drift in fragmented populations (Aguilar et al., 2008), a phenomenon observed in other species where fragmentation constrains dispersal (Browne and Karubian, 2018; Yan et al., 2019; Xie, 2017; Lai, 2021).

Although the differentiation pattern showed a strong positive correlation between genetic and geographical distances, indicating that isolation-by-distance is a major determinant, the results of the Multiple Matrix Regression with Randomization (MMRR) analyses indicated that genetic distance was influenced by both geographical and environmental distances (R²= 0.343, *p* < 0.01). Significantly, isolation-by-distance (R²= 0.423, *p* < 0.01) had a notable impact on genetic distance. The sub-populations of *C. chekiangoleosa* in FS, DSY, and LHG showed gene exchange. Due to geographical distance, these sub-populations were distinct from the other six sub-populations. This phenomenon can account for the findings of the previous genetic structure analysis and principal component analysis. Meanwhile, the MEMGENE analysis reveals that the three sub-populations, where circles of similar size and color represent individuals with comparable scores (**Figure 5A**), also display significant genetic disparities compared to the other six sub-populations.

The genetic differentiation shows a perfect match with the patterns found in other oil - tea camellias, as previously reported by Yan et al. in 2022 and Huang in 2013. Nevertheless, beyond merely characterizing the genetic differentiation of *Camellia chekiangoleosa*, this study also investigated the impact of landscape elements, specifically topographic features, on its genetic differentiation. This offers comprehensive understandings of the causes of this differentiation. After meticulous field investigations and in-depth analyses, it is hypothesized that topographical features, especially mountain ridges, serve as substantial barriers to gene flow. The MEMGENE analysis further identified a distinct spatial genetic structure, which was molded by the ridges and valleys that demarcate Mount Ruozhushan (RZS), Mount Zhushan (ZS), and Mount Shitoushan (STS). The results of the MEMGENE analysis demonstrate that the ZS and STS sub-populations situated on either side of the valley are denoted by black circles, whereas the RZS sub-population separated by a ridge is represented by white circles (**Figure 5B**). This finding suggests that ridges exert a more substantial influence on the genetic differentiation of *C. chekiangoleosa* than valleys. Presumably, this is because ridges serve as more effective barriers to pollinator movement. In contrast, valleys present relatively weaker impediments, which we hypothesize may be primarily attributed to the gravitational dispersal of seeds. It is expected that the construction of infrastructure on mountain ridges will intensify the existing fragmentation and increase the level of genetic differentiation.

In summary, although *C. chekiangoleosa* currently retains a high degree of genetic diversity, its sub-populations remain susceptible to the adverse impacts of habitat loss and fragmentation. Hence, it is of utmost urgency to implement conservation measures centered around the preservation of landscape connectivity. These measures are indispensable for safeguarding the species’ evolutionary resilience.

## Data Availability

The data presented in this study have been deposited in the Genome Variation Map of the China National Center for Bioinformation, with the accession number GVM001365. The dataset can be accessed through the persistent web link: https://bigd.big.ac.cn/gvm/getProjectDetail?Project=GVM001365.
